# Dimensionality and Compositional Effects on Sr–Fe-Based Ruddlesden–Popper Oxides for Oxygen Catalysis

**DOI:** 10.1021/acs.chemmater.5c03458

**Published:** 2026-03-21

**Authors:** Marianela Gómez-Toledo, Ulises Amador, M. Elena Arroyo-de Dompablo

**Affiliations:** † Departamento de Química Inorgánica, Facultad de Ciencias Químicas, 16734 Universidad Complutense de Madrid, 28040 Madrid, Spain; ‡ CEU Universities, Facultad de Farmacia, Departamento de Química y Bioquímica, Urbanización Montepríncipe, Universidad San Pablo-CEU, Boadilla del Monte, 28668 Madrid Spain

## Abstract

Understanding how structural and compositional features influence the Oxygen Reduction Reaction (ORR) and Oxygen Evolution Reaction (OER) in oxygen electrocatalysis is crucial for the rational design of efficient catalysts. The O p-band center, obtained from density functional theory (DFT) calculations, serves as a predictive electronic descriptor linking the composition and structure of the oxide catalyst to ORR and OER activity. Ruddlesden–Popper oxides Sr_*n*+1_Fe_*n*_O_3*n*+1_ (1 < *n* < ∞) provide a versatile platform for tuning this descriptor. Here, we systematically evaluate the effects of dimensionality, Fe substitution, and oxygen nonstoichiometry in the Sr_*n*+1_Fe_*n*(1–*x*)_M_*nx*_O_3*n*+1−δ_ series (*n* = 1, 2, ∞; M = 3d-metal; *x* = 1/8; δ = 0, 1/8). Both increasing slab thickness (*n* = 1 → ∞) and Fe substitution with more electronegative transition metal elements enhance metal–oxygen hybridization, shifting the O p-band center toward the Fermi level by up to 0.2 and 0.45 eV, respectively, whereas 12% oxygen deficiency shifts it downward by up to 0.45 eV. Across the series, the combined effects of composition and structure span a ∼0.7 eV range in the O p-band center, implying only modest intrinsic variations in ORR/OER activity, often surpassed by extrinsic factors such as morphology and microstructure.

## Graphical abstract

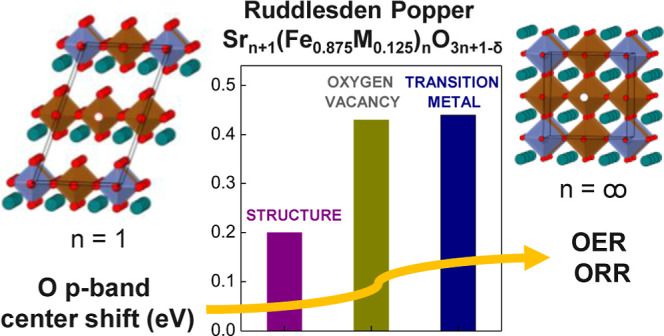

## 1 Introduction

The development of efficient and durable oxygen catalysts is a critical challenge in advancing sustainable energy technologies including water electrolysis, rechargeable metal–air batteries, and regenerative fuel cells. Extensive efforts have been dedicated to the design of novel oxide-based materials that can serve as active, stable, and earth-abundant oxygen electrocatalysts.^1–3^ Despite significant progress, the catalytic performance of many state-of-the-art materials remains insufficient for large-scale applications, highlighting the need for advanced compositions and structure–property paradigms that can guide the discovery of next-generation oxygen catalysts. Among oxide families, perovskite-related structures such as Ruddlesden–Popper (RP) phases have attracted growing attention.^2^ These materials, with general formula A_*n*+1_B_*n*_O_3*n*+1_, consist of perovskite-like blocks of corner-sharing BO_6_ octahedra separated by rock-salt-type AO layers (Figure 1).^4^ By varying the structural parameter *n*, RP oxides offer a platform to systematically tune the dimensionality of the B–O network from quasi-two-dimensional (low *n*) to fully three-dimensional (as *n* → ∞). Several RP systems have already demonstrated promising activity for the Oxygen Reduction Reaction (ORR), and the Oxygen Evolution Reaction (OER), including La_0.5_Sr_1.5_Ni_1–*x*_Fe_*x*_O_4 ±__δ_, Sr_*n*+1_Fe_*n*_O_3*n*+1−δ_, or Sr_*n*+1_(Co_0.8_Fe_0.1_Nb_0.1_)_*n*_O_3*n*+1−δ_, underscoring the central role of dimensionality in governing oxygen electrocatalysis.^1,5–8^ Together with dimensionality, the tunable composition of A_*n*+1_B_*n*_O_3*n*+1_ has profound implications for the electronic structure, charge transport, and defect chemistry, all of which are intimately linked to catalytic behavior. However, a systematic understanding of how composition and dimensionality jointly influence these properties remains an open challenge in rational catalyst design.

**Figure 1:**
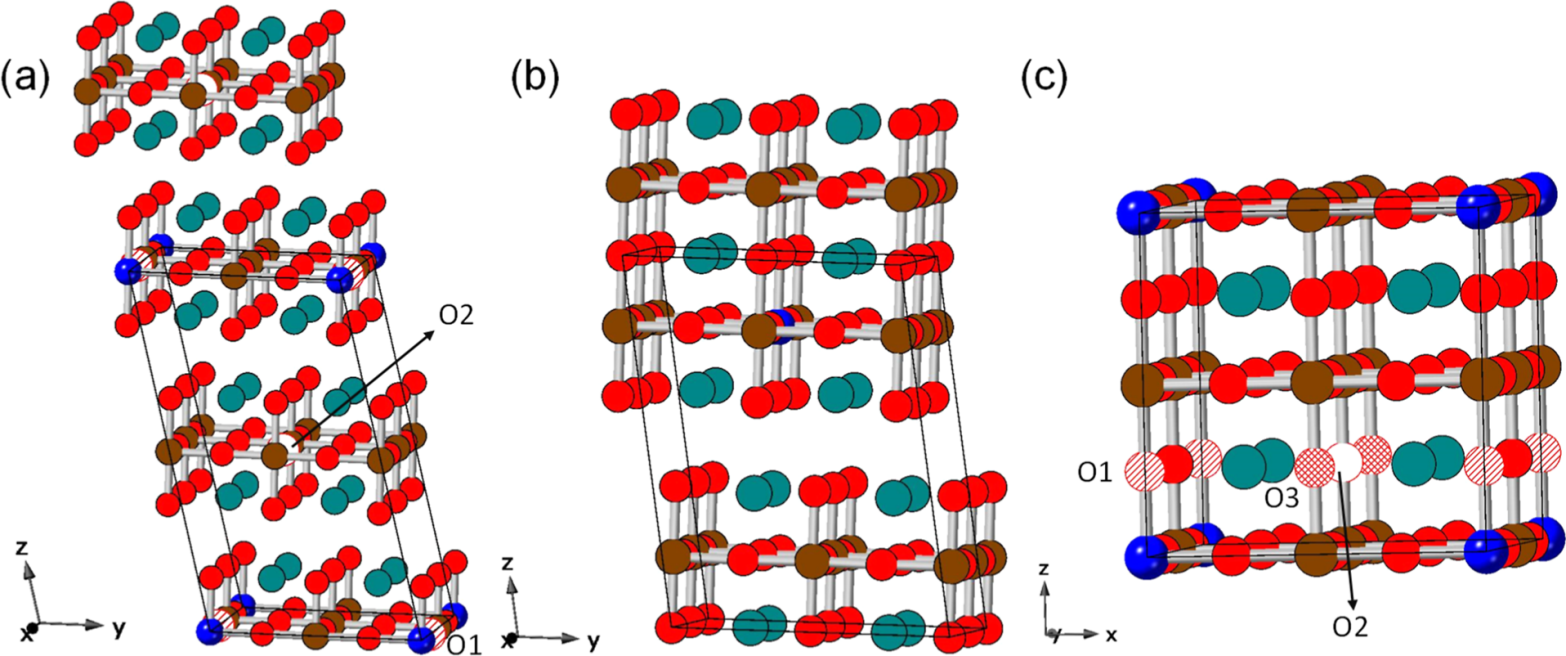
Crystal structures of the supercells used to model Sr_*n*+1_(Fe_0.875_ M_0.125_)_*n*_ O_3*n*+1_ (M = 3d metal): (a) Sr_2_Fe_7/8_M_1/8_O_4_, (b) Sr_3_Fe_14/8_M_2/8_O_7_, and (c) SrFe_7/8_M_1/8_O_3_. O1, O2, and O3 denote the sites for oxygen-vacancy incorporation. Color code: Fe in brown, M in blue, Sr in green, and O in red.

Density functional theory (DFT) has become indispensable for establishing structure–composition–activity relationships in oxygen catalysts.^9^ Electronic descriptors derived from the projected density of states—particularly the O p-band center relative to the Fermi level—have emerged as robust indicators of catalytic potential, correlating with overpotential, oxygen-exchange kinetics, and oxygen-vacancy formation energies.^10–14^ Systematic evaluation of the O p-band center across RP compounds of different dimensionality and composition can therefore provide insight into their catalytic suitability and enable predictive guidelines for designing more efficient oxygen electrocatalysts. With this aim, the present work focuses on the RP-family Sr_*n*+1_Fe_*n*_O_3*n*+1_,^15–17^ a series of materials of interest for oxygen electrocatalysis. The perovskite end-member (*n* = ∞), SrFeO_3‑δ_ is a mixed ionic–electronic conductor which demonstrated potential for the ORR at high temperature and the OER in alkaline media and at high temperature.^18–20^ Its performance can be further improved through low-level Fe-substitution in SrFe_1–*x*_M_*x*_O_3−δ_ (*x* < 0.25).^18,21–24^ This study includes the lower dimensional RP members Sr_2_FeO_4_ (*n* = 1) and Sr_3_Fe_2_O_7_ (*n* = 2), that also show promise for electrocatalytic applications, although the number of available experimental and theoretical studies remains considerably more limited.^1,25–27^

The Sr_*n*+1_Fe_(1–*x*)*n*_M_*xn*_O_3*n*+1_ (*n* = 1, 2, ∞) phases provide a well-defined structural platform to investigate how dimensionality and composition influence the energy position of the O p-band center and, by extension, the material’s activity for oxygen catalysis. To independently evaluate the effect of chemical composition, Fe is partially substituted with a small fraction (1/8) of a 3d transition metal (TM), yielding Sr_*n*+1_ Fe_(1–1/8)*n*_ M_*n*/8_O_3*n*+1_ (M = 3d metal), preserving the formal oxidation state of the B-site cations while introducing modifications to the electronic structure. In addition, the impact of oxygen nonstoichiometry is examined for the *n* = 1 and *n* = ∞ members, by introducing oxygen vacancies (SrFe_7/8_M_1/8_O_2.875_, Sr_2_Fe_7/8_M_1/8_O_3.875_) and evaluating their effect on the O p-band center. Altogether, this approach enables a systematic evaluation of how structural dimensionality and targeted chemical tuning govern the electronic descriptors relevant for oxygen electrocatalysis.

## 2 Methodology

Calculations were performed using the Vienna ab initio simulation program (VASP).^28,29^ The interaction of core electrons with the nuclei is described by the Projector Augmented Wave (PAW) method.^30^ Previous investigations have shown that the Strongly Constrained and Appropriately Normed (SCAN) meta-GGA exchange–correlation functional^31^ correctly reproduces the structural, magnetic, and electronic properties of complex oxides,^32–35^ and it is therefore used in this study. Moreover, Jacobs et al. reported that SCAN yields lower errors than other functionals when comparing the predicted O p-band center values with the actual X-ray emission spectroscopy (XES) O p-band center.^12^ The energy cutoff for the plane wave basis set was set to 600 eV throughout the calculations. The integration in the Brillouin zone was done on the appropriate *k*-point meshes determined by the Monkhorst–Pack scheme. Parameters for the calculations are listed in Table S1.

The initial crystal structures of Sr_2_FeO_4_ and Sr_3_Fe_2_O_7_ were obtained, respectively, from ICSD entries 69849 ^15^ and 74437,^16^ both crystallizing in the *I*4/*mmm* space group. To model the M-substituted compounds, the tetragonal *I*4/*mmm* symmetry was reduced to triclinic. Specifically, a 2 × 2 × 2 supercell of the primitive unit cell was used for the *n* = 1 RP phase (Sr_16_Fe_8_O_32_), while a 2 × 2 × 1 supercell was employed for the *n* = 2 phase (Sr_12_Fe_8_O_28_). In each case, one of the eight Fe atoms was replaced with a dopant M atom, resulting in the compositions Sr_2_Fe_0.875_M_0.125_O_4_ and Sr_3_Fe_1.75_M_0.25_O_7_, respectively (Figure 1a,b). The starting atomic positions for the cubic perovskite SrFeO_3_ were taken from ref 36. A 2 × 2 × 2 supercell of the primitive cubic structure (Sr_8_Fe_8_O_24_, Figure 1c) was constructed, and M substitution was simulated by replacing one Fe atom, yielding SrFe_0.875_M_0.125_O_3._

To calculate the oxygen-vacancy formation energies of Sr_2_Fe_0.875_M_0.125_O_4_ and SrFe_0.875_M_0.125_O_3_, one oxygen atom was selectively removed from the respective supercells Sr_16_Fe_7_MO_32_ and Sr_8_Fe_7_MO_24_. This procedure yields the oxygen-deficient compositions Sr_16_Fe_7_MO_31_ (Sr_2_Fe_7/8_M_1/8_O_3.875_) and Sr_8_Fe_7_MO_23_ (SrFe_7/8_M_1/8_O_2.875_); in both cases, the formal oxidation state of B-site cations is +3.75, which enable evaluation of the influence of the M dopant on the vacancy formation energy. Note that for the perovskite series, this level of oxygen vacancies is close to that experimentally observed at RT^37^ (for instance, SrFeO_2.84_ in ref 21 or SrFe_0.9_Cu_0.1_O_2.71_ in ref 22).

The oxygen-vacancy formation processes can be described by the following reactions:

$$n=\infty :{Sr}_{16}{Fe}_{7}{MO}_{32}\to {Sr}_{16}{Fe}_{7}{MO}_{31}+\frac{1}{2}O_{2}$$ 1

$$n=1:{Sr}_{8}{Fe}_{7}{MO}_{24}\to {Sr}_{8}{Fe}_{7}{MO}_{23}+\frac{1}{2}O_{2}$$ 2

From the computed total energies, the oxygen-vacancy formation energies (*E*_ovf_) can be determined as

3 $$E_{ovf}=E\left({Sr}_{16}{Fe}_{7}{MO}_{31}\right)+\frac{1}{2}E\left(O_{2}\right)-E\left({Sr}_{16}{Fe}_{7}{MO}_{32}\right)$$

4 $$E_{ovf}=E\left({Sr}_{8}{Fe}_{7}{MO}_{23}\right)+\frac{1}{2}E\left(O_{2}\right)-E\left({Sr}_{8}{Fe}_{7}{MO}_{24}\right)$$

Here, *E*(O_2_) denotes the total energy of an isolated oxygen molecule in the gas phase, computed in its spin-polarized ground state after full structural relaxation.

Following previous studies on O p-band centers, all materials were simulated as ferromagnetic to ensure a consistent and tractable set of calculations across the investigated oxides.^12,14,38^ The local magnetic moments are taken from the difference between projected electron densities of up and down spins onto a sphere of 1 Å radius. Bader charge analysis^39^ was performed on the charge density files^40^ using the Pymatgen package.^41^

The O p-band center has been calculated as the centroid of the projected density of states (PDOS) of oxygen, including occupied and unoccupied states (up to a maximum energy, *E*_max_), according to eq 5,^11^ where *E* is the electron energy and *D*_Op_ (*E*) is the DOS projected onto the p orbitals of O atoms. The band center is referred to the Fermi level, which in the case of semiconducting oxides is usually set at the conduction band minimum.^13^ Note that bulk Op-band centers correlate, and can be used to describe, the surface O p-band centers^13,42^

$$O\;p-band\;center=\frac{\int_{E_{\min}}^{E_{\max}}ED_{Op}\left(E\right)dE}{\int_{E_{\min}}^{E_{\max}}D_{Op}\left(E\right)dE}-E_{Fermi}$$ 5

The choice of an upper energy, *E*_max_, is ambiguous because the quantity of unoccupied states present in the calculated PDOS depends on the system and the number of bands used in the calculation (Figure S1). A good criterion is to choose *E*_max_ considering how many of the unoccupied states are chemically relevant.^43^ Hence, a reasonable *E*_max_ cutoff would be the vacuum level since above that, the states are actually unstable with respect to the electron leaving the material. Since this is typically around 4 eV for perovskite oxides,^44,45^ this value is adopted as *E*_max_ in the present work.

## 3 Results

In the general formula A_*n*+1_B_*n*_O_3*n*+1_, the B–O framework is quasi-two-dimensional for low *n* values (*n* = 1 and *n* = 2), whereas it becomes fully three-dimensional as *n* → ∞. As dimensionality increases, the stronger orbital overlap leads to enhanced bond hybridization (i.e., covalency).^6,46,47^ As previously noted,^12,13^ the value of the O p-band centers gets closer to the Fermi level with enhanced covalency and, therefore, in the RP series, a shallower O-p band center is expected with increasing dimensionality. Figure 2a–c shows the calculated DOS for the RP-Sr_*n*+1_Fe_*n*_O_3*n*+1_ phases, highlighting the contribution from Fe, O, and Sr states. The Fe and O states are strongly hybridized, forming a broad band that extends from −8 up to approximately 2 eV above the Fermi level. Consistent with the dimensionality argument, the extracted O p-band center for Sr_2_FeO_4_ (−2.96 eV) is below that of the SrFeO_3_ perovskite (−2.85 eV).

**Figure 2:**
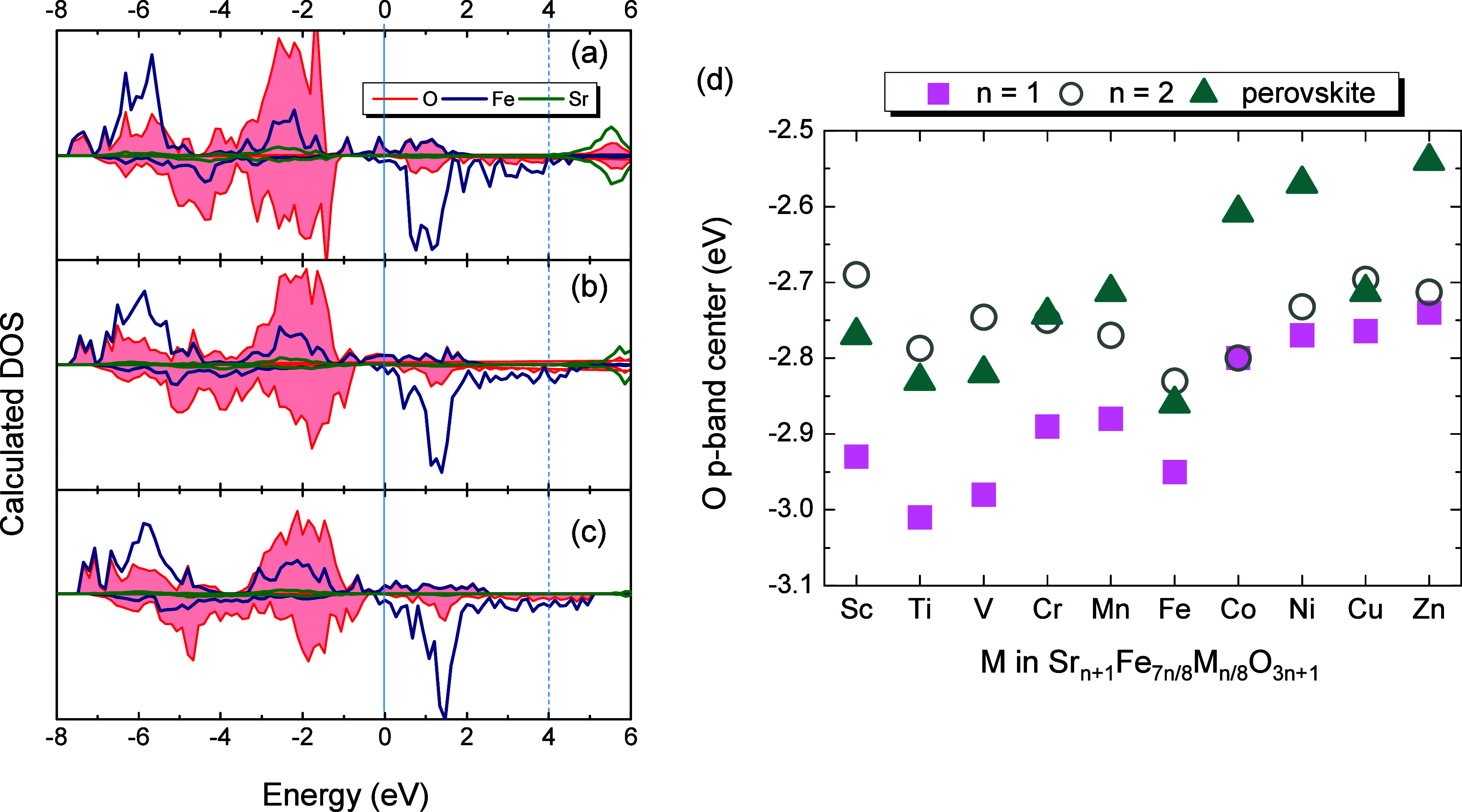
Calculated atom-projected density of states for Sr_*n*+1_Fe_*n*_O_3*n*+1_ with (a) *n* = 1, (b), *n* = 2, and (c) *n* = ∞. The Fermi level is set as the zero of energy. Up spin (or majority) and down spin (or minority) contributions are shown. Color code: Fe: navy, O: red, and Sr: green. (d) O p-band centers of Sr_*n*+1_Fe_7*n*/8_M_*n*/8_O_3*n*+1_. Pink squares, open circles, and green triangles denote, respectively, the *n* = 1, *n* = 2, and *n* = ∞ members.

In simple ABO_3_ perovskites, it is well-established that B–O bond covalency increases across the 3d series, leading to a progressively shallower O p-band center as the 3d electron count increases.^11,12^ Building on this known trend, we investigated how partial substitution of Fe by other 3d-TMs affects the electronic structure of Sr_*n*+1_Fe_*7n*/8_M_*n*/8_O_3*n*+1._ Results show that the expected increase in B–O covalency across the 3d series is preserved (Figure 2d). For a given TM, when the effect of dimensionality is considered, the *n* = 1 RP members systematically exhibit a deeper O p-band center than their perovskite (*n* = ∞) counterparts. However, the *n* = 2 compositions display a distinct and unexpected deviation from this dimensionality-driven trend. Rather than presenting O p-band-center values intermediate between those of the *n* = 1 and perovskite phases, the *n* = 2 members lie above the perovskite values for early 3d-elements, while for late 3d-elements, they more closely resemble the *n* = 1 compounds. This anomalous behavior disappears when the *E*_max_ in eq 5 is extended to include higher-energy unoccupied states (Figure S2). Furthermore, the calculated Bader charges for Fe and O in the *n* = 2 compositions fall between those of the *n* = 1 and *n* = ∞ phases (Figure S3). A comprehensive mechanistic understanding of this distinctive regime for the *n* = 2 members lies beyond the scope of the present work and will be addressed in a dedicated follow-up study.

Oxygen nonstoichiometry is of paramount relevance for the catalytic properties of TM oxides, as it directly governs their defect chemistry, electronic structure, and oxygen transport.^1,44,48^ Regarding defects energetics, perovskite SrFeO_3_ is typically obtained in a nonstoichiometric form (SrFeO_3−δ_), while stoichiometric Sr_2_FeO_4_ can be more easily synthesized.^15,36,49,50^ Accordingly, in this work, the calculated energy of vacancy formation for SrFeO_3_ is of 1.97 eV, consistent with previous reports,^22,23,37^ whereas that of Sr_2_FeO_4_ raises to 2.6 eV. These benchmarks serve as a reference for the subsequent exploration of oxygen-vacancy formation in the Fe-substituted *n* = 1 and *n* = ∞ series.

For Sr_2_FeO_4_, calculations indicate that the removal of equatorial oxygen atoms is energetically more favorable than that of apical oxygen, by approximately 0.5 eV per formula unit.^38^ Therefore, only equatorial oxygen sites are considered for the investigation in M-substituted Sr_2_Fe_7/8_M_1/8_O_4_ oxides. These sites are illustrated in Figure 1a: O1, shared between M and Fe, and O2, coordinated exclusively to Fe. In SrFe_7/8_M_1/8_O_3_ perovskites, there are also oxygen sites shared between Fe and M (labeled as O1) and oxygen sites not bonded to M. For the latter, two distinct chain motifs can be distinguished, O_vac_–Fe–O–Fe and O_vac_–Fe–O–M, which give rise, respectively, to the oxygen vacancies labeled as O2 and O3 in Figure 1c. The calculated oxygen-vacancy formation energies for all compounds are positive (Figure 3a), indicating that their reduction is not thermodynamically favored at 0 K.

**Figure 3:**
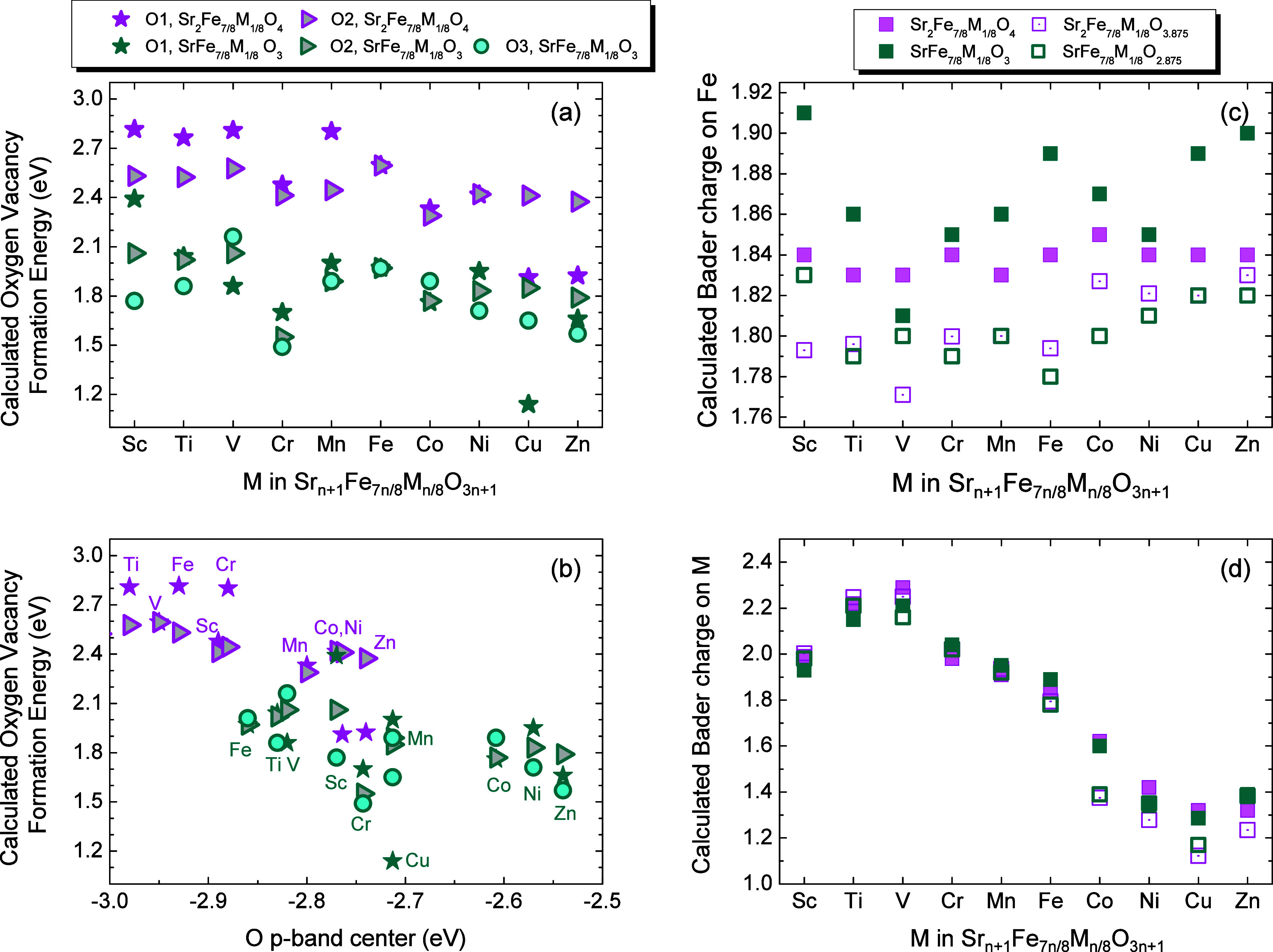
(a) Calculated energy of oxygen-vacancy formation according to the reactions (3) and (4) for Sr_2_Fe_7/8_M_1/8_O_4_ (pink symbols) and SrFe_7/8_M_1/8_O_3_ (green symbols). The different oxygen sites are indicated by asterisks (O1), triangles (O2), and circles (O3). (b) Calculated energy of oxygen-vacancy formation as a function of the O p-band center for Sr_2_Fe_7/8_M_1/8_O_4_ (pink symbols) and SrFe_7/8_M_1/8_O_3_ (green symbols) for the different O-sites. (c,d) Calculated Bader charges for Fe/M ions of Sr_2_Fe_7/8_M_1/8_O_4_ (filled pink) and SrFe_7/8_M_1/8_O_3_ (filled green), compared to their respective oxygen-deficient phases Sr_2_Fe_7/8_M_1/8_O_3.875_ (open pink) and SrFe_7/8_M_1/8_O_2.875_ (open green).

Building on the analysis of the distinct oxygen sites and their local environments, the calculated energies reveal clear trends in oxygen-vacancy formation. It is observed that the formation energy is significantly lower in perovskites than that in the *n* = 1 RP counterparts, highlighting the role of dimensionality in defect energetics. Regarding compositional effects, a clear trend emerges across the 3d-series, with the propensity for oxygen-vacancy formation increasing from early to late TMs by as much as 0.8 eV. This tendency is primarily driven by the progressive weakening of the M–O bond across the series,^51^ coupled to the stability of the lower oxidation states of the TM cations, which energetically favor vacancy formation. The differences in the formation energies for the distinct oxygen sites for a given TM and structure range from a minimum of 0.05 eV for Co (*n* = 1) to a maximum of 0.62 eV for Sc (*n* = ∞). In general, consistent with expectations based on the strength of the Fe–O and M–O bonds being broken, the most favorable sites for early TMs are the O2 or O3 sites, which involve the breaking of two Fe–O bonds, whereas for late TMs, the O1 site, involving the breaking of one Fe–O and one M–O bond, becomes more favorable. Additionally, for SrFe_7/8_Cu_1/8_O_3_ and SrFe_7/8_V_1/8_O_3_ perovskites, the tendency of Cu and V to adopt 5-fold coordination may further promote oxygen-vacancy formation at the O1 site. These general trends are further confirmed by the calculated formation energies for late 3d TMs, which are in good agreement with those of previous works. Specifically, the reported formation energy of SrFeO_3_ decreases from 2.0 to 0.90 eV in Cu-doped SrFe_0.725_Cu_0.25_O_3_,^22^ and from 2.1 to 1.85 eV^23^ and 1.7 eV,^52^ in SrFe_7/8_Co_1/8_O_3_ and SrFe_7/8_Ni_1/8_O_3_, respectively. Furthermore, the previously observed correlation between oxygen-defect formation energies and O p-band centers in ABO_3_ perovskites^10^ and (La_1–*x*_Sr_*x*_)_2_MO_4±δ_ (M = Mn, Co, Ni, and Cu)^53^ is also noted in the present case (Figure 3b). In general, oxides with shallower O p-band centers exhibit a higher propensity for oxygen-vacancy formation.^13^

Oxygen vacancies induce an electronic density redistribution that leads to the reduction of cations to lower oxidation states. In both Sr_2_Fe_7/8_M_1/8_O_4_ and SrFe_7/8_M_1/8_O_3_, the formal oxidation state at the B site (i.e., for Fe and M) is +4 in the stoichiometric oxides and +3.75 in the oxygen-deficient models. To assess the effect of oxygen vacancies, Figure 3c,d compares the calculated Bader charges of M and Fe for the most stable structures with vacancies (open squares) and without vacancies (filled squares). The general trend is that introducing oxygen vacancies decreases the Bader charge on Fe, indicating Fe reduction, with the effect being more pronounced in the perovskite structure. Previous studies have debated whether, in SrFeO_3−δ_, the reduced Fe ions reside in square-pyramidal or octahedral coordination environments.^36,54^ In the present calculations for perovskites, the reduction is localized on the 5-fold-coordinated Fe ions. As expected, for the late 3d elements, the charge-density redistribution affects both Fe and M, consistent with the tendency of these TM to adopt lower oxidation states. The SrFe_7/8_V_1/8_O_3−δ_ perovskites are an interesting case. In the stoichiometric phase, the calculated magnetic moments indicate the presence of V^5+^ ions (Figure S3d), while Fe exhibits the most reduced character among the perovskite series (Figures S3 and 3c). Upon oxygen-vacancy incorporation, the formation of V-centered square pyramids leads to a substantial reduction of V^5+^ (Figure 3d) and, concurrently, the smallest change in Fe charge compared to that of the other perovskites (Figure 3c).

Figure 4a shows the O p-band centers extracted for the most stable oxygen-deficient models. As in the stoichiometric oxides, increasing dimensionality shifts the O p-band center closer to the Fermi level. In addition, when compared to the stoichiometric phases, the oxygen-deficient oxides exhibit a clear downshift of the O p-band center (Figures 4a and S4–S5). This behavior can be rationalized by the reduction of the B-site cations induced by oxygen-vacancy formation, which diminishes the Fe-3d/O-2p hybridization, reduces the covalent character of the Fe–O bond, and consequently lowers the O p-band center relative to the Fermi level.^53,55^

**Figure 4:**
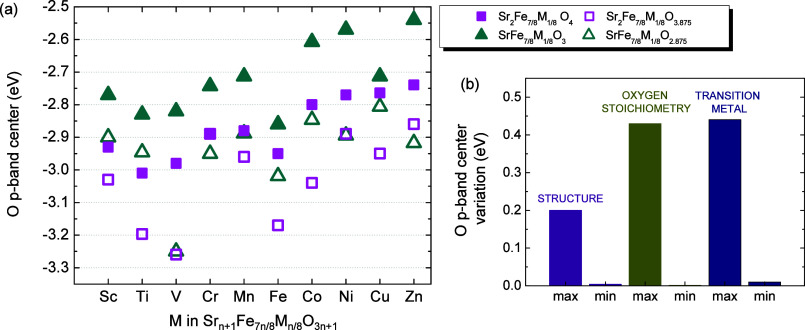
(a) The O p-band centers of Sr_*n*+1_Fe_7*n*/8_M_*n*/8_O_3*n*+1−δ_ extracted using *E*_max_ = 4 eV in eq 5 of the main text. Filled symbols denote the oxygen-stoichiometric compounds, while open symbols correspond to their oxygen-deficient counterparts (*n* = 1 pink squares, *n* = ∞ green triangles). For the oxygen-deficient phases, only the O p-band center associated with the most stable oxygen-vacancy configuration is shown. (b) Range of O p-band center values shown in panel (a), with bars indicating the maximum and minimum variation due to structure (purple), oxygen content (olive), and TM (blue).

## 4 Discussion

In Sr_*n*+1_Fe_*7n*/8_M_*n*/8_O_3*n*+1−δ_, both structure (dimensionality, *n*) and composition (nature of M and oxygen content) influence the O p-band center. From an application standpoint, bringing the O p-band center closer to the Fermi level enhances catalytic activity, as reflected by lower area-specific resistance (ASR) in high-temperature ORR measurements in solid oxide systems and by reduced overpotentials for the OER in aqueous electrolytes.^10,11,13,14^ Following this guiding principle, a quick inspection of Figure 4a shows that the most active Sr_*n*+1_Fe_*7n*/8_M_*n*/8_O_3*n*+1−δ_ compounds are perovskites containing late 3d-TM with a high oxygen content, whereas the least active materials correspond to the *n* = 1 phases incorporating early TMs and oxygen deficiency. Across the full data set, the O p-band center spans from −2.6 eV for Sr_2_Fe_7/8_Zn_1/8_O_3_ (an unrealistic material) to −3.3 eV for Sr_2_Fe_7/8_V_1/8_O_3.875_, yielding a total variation of ∼0.7 eV. Figure 4b disentangles how dimensionality and composition jointly shape this band-center landscape.

For dimensinality, the structural change from *n* = 1 to perovskite generally induces a variation in the band center, on the order of ∼0.2 eV (e.g., SrFe_7/8_Ti_1/8_O_2.875_ vs Sr_2_Fe_7/8_Ti_1/8_O_3.875_), presuming a higher activity as dimensionality increases. Benchmarking with experiments is difficult, since systematic investigations of dimensionality are scarce in the literature, in part due to the difficulties in the synthesis of the single phase for some RP phases.^49,56–58^ Cao et al. succeeded to prepare La_*n*_SrNi_*n*_O_3*n*+1_ (*n* = 1, 2, 3, ∞), where Ni adopts a formal oxidation state of +3. These nickelate oxides displayed a remarkable enhancement in the OER activities as the dimensionality increased with *n*.^6^ While this supports the general trend observed in Figure 4, it should be remarked that for some oxygen-deficient compounds (e.g., SrFe_7/8_Ni_1/8_O_2.875_ vs Sr_2_Fe_7/8_Ni_1/8_O_3.875_), there is no difference in O p-band center between *n* = 1 and *n* = ∞. Therefore, one cannot categorically claim that perovskites are intrinsically more active than *n* = 1 phases; intrinsic activity depends critically on chemical composition. Indeed, according to the O p-band centers, the oxygen-stoichiometric *n* = 1 phases with M = Cu or Ni could outperform the intrinsic activity of oxygen-deficient perovskites with M = Ti or V.

The DFT results evidence that perovskites form oxygen vacancies more readily than their *n* = 1 counterparts (Figure 3a,b). Oxygen vacancies are crucial for the ORR at high temperature, as they promote faster oxygen adsorption, dissociation, and incorporation into the lattice, thereby enhancing overall electrocatalytic performance.^59^ The greater tendency of perovskites to accommodate oxygen vacancies, as revealed by the present DFT calculations, is consistent with prior experimental reports showing that oxygen-deficient perovskites often display enhanced catalytic activity, albeit accompanied by larger thermal expansion and reduced structural stability compared to RP phases.^1,18^ Moreover, in perovskites with high oxygen-vacancy concentrations and strong metal–oxygen covalency, lattice oxygen might directly participate in O_2_ evolution from alkaline electrolytes, in the so-called lattice oxygen mechanism (LOM). In this mechanism, which operates alongside the conventional adsorbate evolution mechanism (AEM), water reacts with lattice oxygen instead of metal sites, and oxygen vacancies are formed while O_2_ is generated.^5,8,55^ At the same time, it is important to note that the LOM is not universal for perovskites; its activation depends on the nature of the TM and oxygen content. To date, only a few catalysts have been reported to adhere to the LOM, and in particular, Sr_*x*_Ca_1–*x*_FeO_3−δ_ perovskites are found to evolve oxygen predominantly via the conventional AEM.^60^ Figure 3a,b reveals that the LOM in iron-based perovskites could be enhanced if Fe is partially replaced by more electronegative TM elements. Interestingly, some *n* = 1 members containing late TM elements exhibit competitive oxygen-vacancy formation energies, comparable even to some perovskites. This suggests that the LOM pathway may also be activated in low-dimensional RP terms, as has indeed been demonstrated for RP-Sr_3_(Co_0.8_Fe_0.1_Nb_0.1_)_2_O_7−δ_ ^8^ and La_0.5_Sr_1.5_Ni_1–*x*_Fe_*x*_O_4±δ_.^5^

As shown in Figure 4 , oxygen content is a key determinant of the electronic structure, shifting the O p-band center by up to 0.44 eV in some cases (e.g., SrFe_7/8_V_1/8_O_3_ vs SrFe_7/8_V_1/8_O_2.875_), while in others, its effect is minimal (e.g., Sr_2_Fe_7/8_Cr_1/8_O_4_). Likewise, the identity of M can vary the band center by as much as 0.44 eV (SrFe_7/8_V_1/8_O_2.875_ vs SrFe_7/8_Cu_1/8_O_2.875_) and as little as 0.01 eV (e.g., Sr_2_Fe_7/8_Cr_1/8_O_4_ vs Sr_2_Fe_7/8_Mn_1/8_O_4_). Importantly, these factors are interdependent as the nature of M largely dictates the achievable oxygen content. Cations stable at low oxidation states (e.g., Co^2+^, Cu^2+^) rarely form stoichiometric perovskites, whereas high-valence cations (e.g., V^5+^, Mo^6+^) favor perovskites with fewer oxygen vacancies.

It is legitimate to question whether the variations of the O p-band center identified in this work have quantitative repercussions on catalytic activity. To address this, one can examine the correlations reported between DFT-O p-band centers and measured catalytic activities in the OER and ORR. It is important, however, to emphasize that catalytic activity measurements inevitably reflect a convolution of intrinsic catalyst properties and extrinsic factors such as the morphology, microstructure, and electrode architecture. Consequently, the following discussion should be interpreted primarily in terms of semiquantitative trends rather than absolute catalytic metrics. For the OER in alkaline solution, although the O p-band center is a well-established electronic descriptor and numerous perovskite oxides have been studied experimentally, a direct and systematic correlation between this descriptor and measured overpotentials remains largely unexplored. Nonetheless, in the study of (Ln_0.5_Ba_0.5_)­CoO_3−δ_ double perovskites, a shift of the O p-band center by ∼0.5 eV corresponds to a modest change of ∼0.07 V in overpotential.^14^ A similar O p-band center shift upon fluorine incorporation in La_0.5_Ba_0.5_Co_3–*x*_F_*x*_ (*x* = 0, 0.1) translates into a measured OER overpotential difference of 0.05 V at 100 mA/cm^2^.^61^ More combined experimental and computational investigations are needed to assess the relationship between the O p-band center and catalytic activity in aqueous solutions.

In contrast, at high temperature, a more comprehensive correlation has been demonstrated and a database covering dozens of perovskites shows a statistically significant linear relationship (*R* ^2^ ≈0.86) between the bulk O p-band center and the logarithm of the surface exchange coefficient (k*).^10–12^ According to the linear relationship reported by Morgan et al.,^11^ the O p-band centers observed for realistic materials in our systems (Table S2) would yield only moderate variations of less than two factors in k*. While such variations are not negligible in principle, their impact becomes minor once extrinsic factors such as porosity, particle size, layer thickness, surface reconstruction, and other morphological parameters are considered. Moreover, when RP oxides are employed as very thin layers or films, substrate-induced effects such as epitaxial strain or interfacial interactions may further influence the catalyst performance, an aspect not explicitly addressed in the present bulk DFT analysis. These extrinsic factors are critical in solid oxide cell devices (fuel cells, SOFCs; and electrolyzers, SOECs) working at intermediate to high temperatures with gases as reactants and products. Indeed, several studies report quantitative improvements due to microstructural and morphology optimization. It is very common in the literature to report the catalytic properties of composite electrodes consisting of the material under study and electrolyte in different proportions. In addition, various strategies are used both to synthesize the materials and to prepare the composites to ensure high specific surface area and high porosity. This maximizes the triple phase boundary (TPB) (contact surface between O_2_ molecules, electrocatalytic material, and electrolyte through which oxide anions must diffuse) where the ORR and OER take place. Sophisticated strategies can be employed, such as the construction of electrodes by single-step spray pyrolysis with simultaneous deposition of the catalyst and electrolyte and even decoration with metal nanoparticles.^18,21,62–64^

Taken together, the above examples suggest that in the Sr_*n*+1_Fe_*n*(1–*x*)_M_*nx*_O_3*n*+1−δ_ series (*n* = 1, 2, ∞; M = 3d metal; *x* = 1/8; δ = 0,1/8), extrinsic factors can induce catalytic activity enhancement comparable to, or even larger than, those expected from electronic tuning alone (e.g., modest shifts in the O p-band center). Therefore, while compositional tuning and its effect on the O p-band center may contribute to performance of the extensively studied Sr–Fe–O-based RP series, morphology, microstructure, and electrode conformation are likely to play an equal, or even dominant, role.

In the present study, the focus is on bulk electronic descriptors for catalytic activity, while operando surface evolution and long-term stability remain open questions for further investigation. Future work could extend the present DFT analysis by considering the behavior of these oxides under realistic OER/ORR conditions, where surface reconstruction or partial amorphization may influence the structure of the catalytically active region.^65^ Moreover, the O p-band center represents only one of several relevant descriptors of electrocatalytic activity.^9,42^ Correlating this bulk descriptor with surface-specific quantities, such as the adsorption energies of key OER/ORR intermediates, and performing explicit computational surface studies, therefore represents an important avenue for future work to better assess the catalytic potential of this family of oxides.^66^

## 5 Conclusions

Systematic evaluation of the O p-band center of the RP-Sr_*n*+1_Fe_7*n*/8_M_*n*/8_O_3*n*+1−δ_ series provides insight into their catalytic potential and enables predictive guidelines for designing more efficient oxygen electrocatalysts. Both 12% Fe-substitution by late 3d-TM and increasing dimensionality (*n* → ∞) enhance B–O covalency, yielding shallower p-band and often lower oxygen-vacancy formation energies. The oxygen content can shift the O p-band center by up to 0.44 eV, comparable to the effect of Fe-substitution by 12% TM, showing that oxygen stoichiometry is a critical element of composition–activity relationships. Because the nature of the substituent –TM dictates the achievable oxygen stoichiometry, electronic tuning via B-site substitution cannot be decoupled from defect chemistry.

Defect energetics further distinguish between *n* = 1 and perovskite structures. Perovskites form oxygen vacancies more readily, suggesting enhanced ORR/OER activity. Noteworthily, certain *n* = 1 compositions with late 3d metals exhibit vacancy formation energies close to those of perovskites, indicating that layered RP phases can also reach vacancy-rich regimes compatible with LOM pathways.

The electronic trends align with the heuristic that shallower p-band O centers favor enhanced electrocatalytic activity; however, literature correlations indicate that the ∼0.7 eV range spanned by the entire RP system yields only modest activity gains. These improvements can easily be overshadowed by extrinsic factors—such as morphology, microstructure, porosity, and surface area—that may induce equal or larger changes in measured performance. Overall, dimensionality, partial Fe-substitution, and oxygen content provide meaningful but moderate intrinsic levers for tuning the activity in Sr–Fe–O-based RP materials. These findings underscore that rational catalyst design must integrate electronic-structure engineering with careful morphological and microstructural control to unlock the activity in oxygen electrocatalysis.

## Supplementary Material



## Notes

Details of the computational method, analysis of O p-band centers, calculated Bader charges, local magnetic moments, and DOS (PDF)
