# Supplementary material for: Dimensionality and Compositional Effects on Sr–Fe-Based Ruddlesden–Popper Oxides for Oxygen Catalysis
Source: Chem Mater. 2026 Mar 21;38(7):3572–80. doi: 10.1021/acs.chemmater.5c03458 (PMC13086125; doi:10.1021/acs.chemmater.5c03458)
Supplement: Supplementary file 1 [file cm5c03458_si_001.pdf]

Supporting Information

Dimensionality and Compositional Effects on Sr-Fe-based Ruddlesden–Popper Oxides for Oxygen Catalysis

Marianela Gómez-Toledo[1], Ulises Amador[2] and M. Elena Arroyo-de Dompablo[1*]

[1]Departamento de Química Inorgánica, Facultad de Ciencias Químicas, Universidad Complutense de Madrid, 28040 Madrid, Spain

[2]CEU Universities, Facultad de Farmacia, Departamento de Química y Bioquímica, Urbanización Montepríncipe, Universidad San Pablo-CEU, Boadilla del Monte, Madrid E-28668, Spain

*Correspondence: e.arroyo@quim.ucm.es

Figure S1: Dependence of O p-band center with calculation parameters

Table S1: Details for the computational method

Figure S2: O p-band center for stoichiometric $Sr_{n+1}Fe_{7n/8}M_{n/8}O_{3n+1}$ at different E_{max} values

Figure S3: Calculated Bader charges and local magnetic moments for stoichiometric $Sr_{n+1}Fe_{7n/8}M_{n/8}O_{3n+1}$

Figure S4: O p-band centers for the distinct oxygen-vacancy sites in oxygen-deficient $Sr_{n+1}Fe_{7n/8}M_{n/8}O_{3n+1-\delta}$

Figure S5: Calculated DOS of $SrFeO_3$ and $SrFeO_{2.875}$

Table S2: O p-band centers and predicted surface exchange coefficient

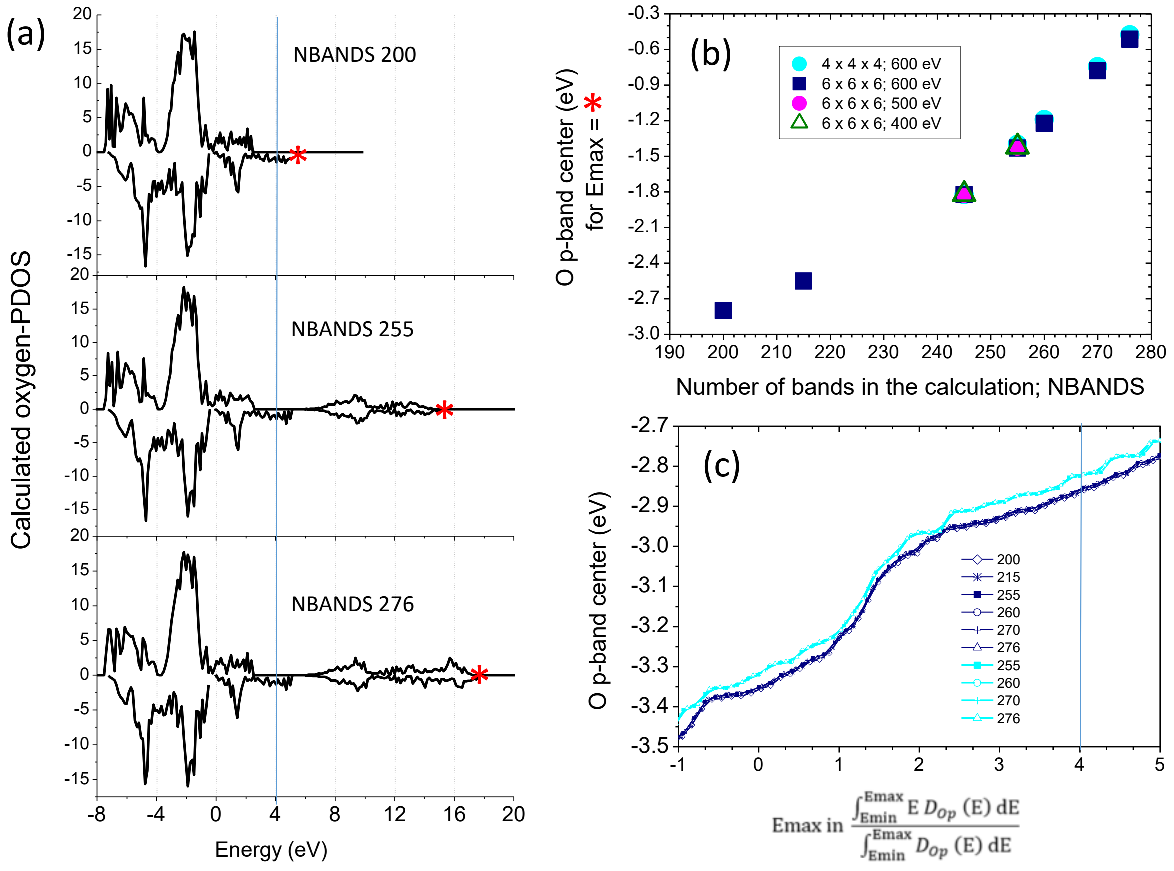

Figure S1. Dependence of O p-band center with calculation parameters.(a) Calculated density of states projected (PDOS) onto the p-orbitals of O atoms for $SrFeO_3$ using different number of bands (NBANDS), a *k*-point mesh 6 x 6 x 6 and a plane-wave energy cutoff of 600 eV. The Fermi level is set as the zero of energy. It is evident that NBANDS is crucial for the quantity of unoccupied states. Above the energy marked by the red asterisk, no additional unoccupied states appear in the PDOS. This energy corresponds to the $E_{max}\rightarrow\infty$ limit in Eq. (5) in the main text:

$$O\ 2p - \text{band center} = \frac{\int_{Emin}^{Emax} E\, D_{O2p}\,(E)\, dE}{\int_{Emin}^{Emax} D_{O2p}\,(E)\, dE} - E_{Fermi}$$

(b) O p-band center extracted from calculations performed with different NBANDS values, and considering $E_{max}\rightarrow\infty$ limit in Eq. (5) of the main text. The O p-band center gets shallower with the increasing number of bands, i.e. quantity of unoccupied states. In contrast, the O p-band center is almost insensitive to the k-point mesh, and the energy cutoff for plane waves. (c) Variation of the O p-center as a function of the E_{max} value in Eq. (5) of the main text, for calculations with NBANDS ranging from 200 (diamonds) to 276 (triangles). At a given energy within the depicted interval, the O p-band center value is independent of the number of bands used in the calculations. The vertical line indicates the E_{max} selected throughout this work (4 eV). Cyan and blue markers denote the 4 x 4 x 4 and 6 x 6 x 6 k-point meshes, respectively.

Table S1. Details for the computational method. The columns list the number of bands (NBANDS), number of ions (NIONS), k-point meshes (KPOINTS), and the number of formula units per cell (Z) used in the calculations.

Stoichiometric $Sr_{n+1}Fe_{7n/8}M_{n/8}O_{3n+1}$			
	n = 1	n = 2	n = ∞
NBANDS	336	288	252
NIONS	56	48	40
Z	8	4	8
KPOINTS	6 x 6 x 4	6 x 6 x 4	6 x 6 x 6
Oxygen deficiency in $Sr_{n+1}Fe_{7n/8}M_{n/8}O_{3n+1-\delta}$ (δ = 0, 0.125)			
	n =1		n = ∞
NBANDS	324		240
KPOINTS	4 x 4 x 2		6 x 6 x 6
VASP_PAW_PBE pseudopotentials utilized in the calculations. The number of valence electrons (ZVAL) is given in parentheses			
O 08Apr2002 (6)		Mn_sv 23Jul2007 (15)	
Sr_sv 07Sep2000 (10)		Fe_sv 23Jul2007 (16)	
Sc_sv 07Sep2000 (11)		Co_sv 23Jul2007(17)	
Ti_sv 26Sep2005 (12)		Ni_pv 06Sep2000 (16)	
V_sv 02Aug2007 (13)		Cu_pv 06Sep2000 (17)	
Cr_sv 23Jul2007 (14)		Zn 08Feb2005 (18)	

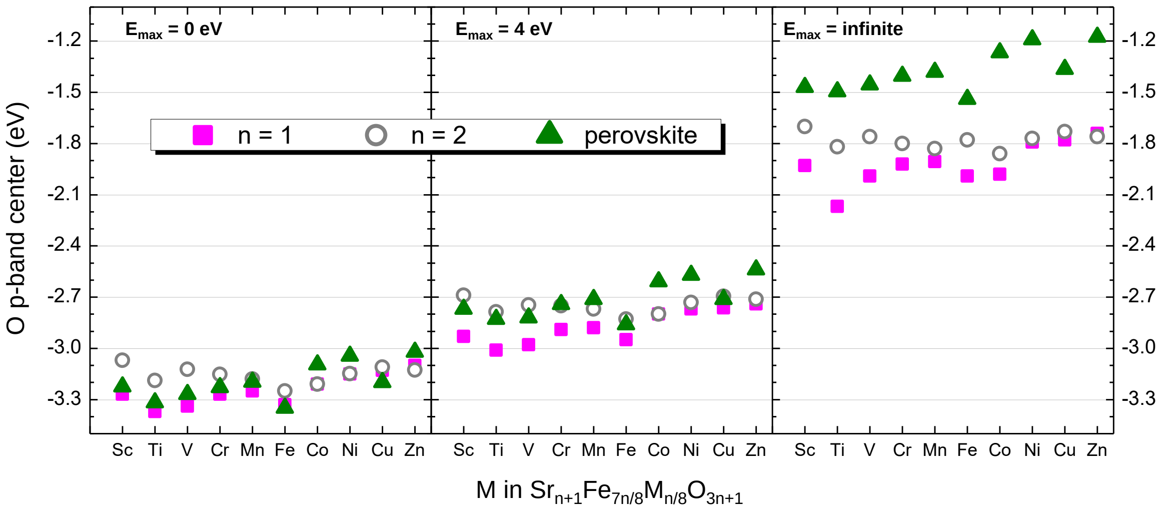

Figure S2. O p-band center position for stoichiometric $Sr_{n+1}Fe_{7n/8}M_{n/8}O_{3n+1}$ taking different E_{max} values in equation (5) of the main text. E_{max} = infinite denotes $E_{max} \rightarrow \infty$ limit, this is, the results considering the raw calculated DOS without discarding any unoccupied state.

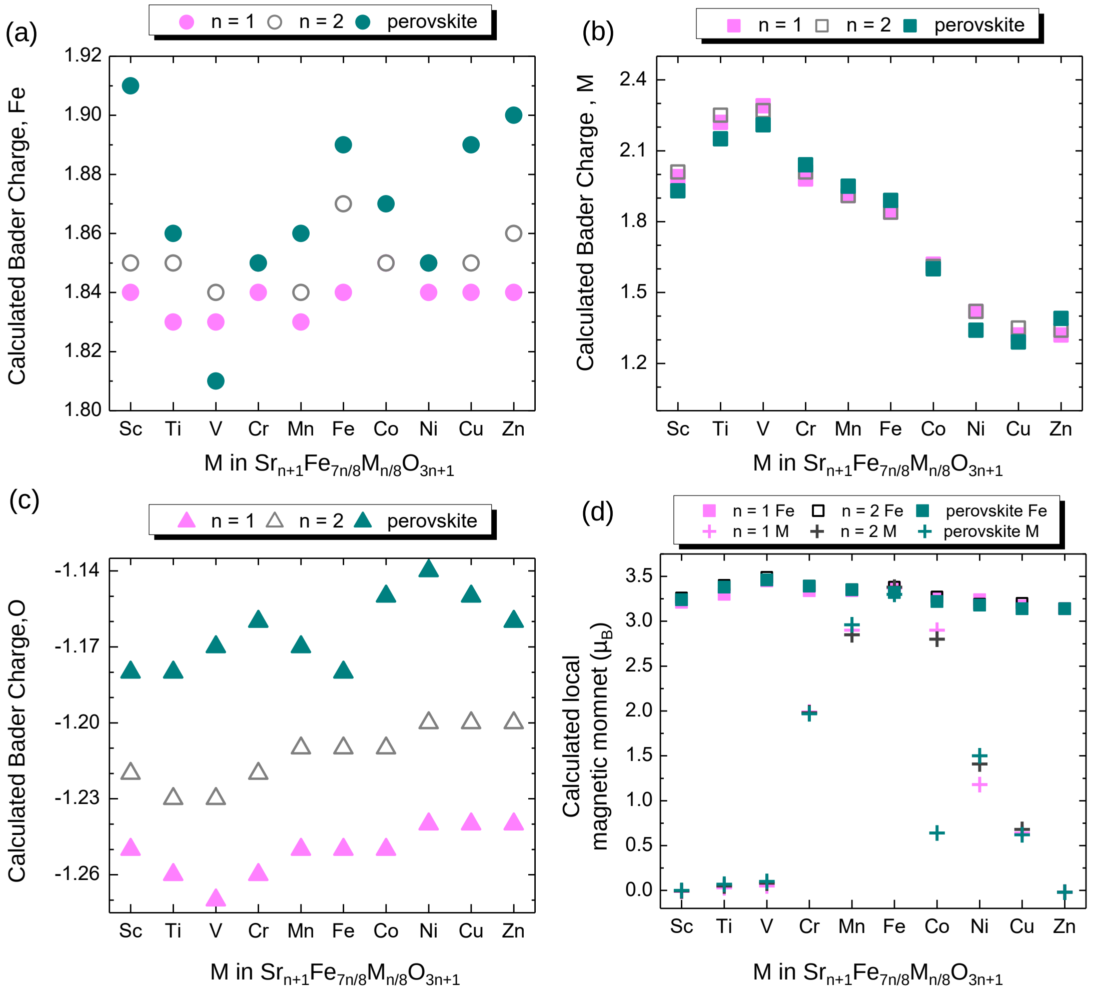

Figure S3. (a-c) Calculated Bader charges for Fe, M and O in $Sr_{n+1}Fe_{7n/8}M_{n/8}O_{3n+1}$. Bader charges on O ions denote an increasing covalency with dimensionality. (d) Local magnetic moments for Fe and TM ions, supporting the increasing covalence of Fe–O bonding moving to the right of the 3d series. While early 3d TMs exhibit values compatible with well-defined oxidation states (Ti^{4+}, V^{5+}, Cr^{4+}), such assignments become increasingly ambiguous for late 3*d* elements (Ni, Cu), where the moments do not correspond clearly to discrete ionic configurations. Altogether, these data suggest that as one moves from left to right across the 3*d* series, the increasing covalency of the TM–O bonds leads to significant electron delocalization, blurring the concept of discrete oxidation states and promoting a more mixed-valence or covalent bonding regime.

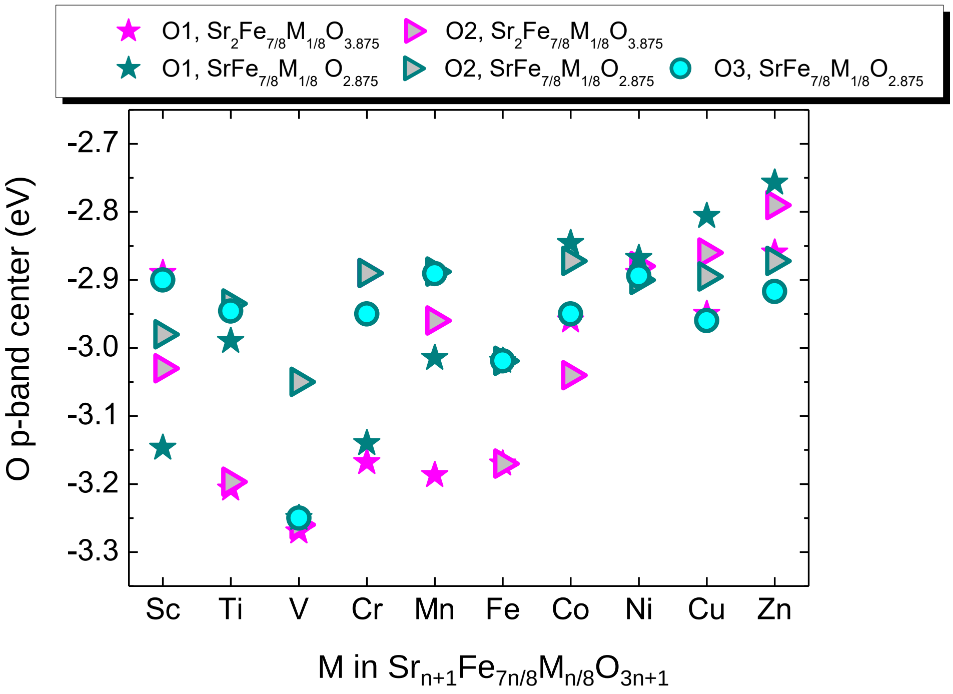

Figure S4. The O p-band centers of $Sr_2Fe_{7n/8}M_{n/8}O_{3.875}$ (pink symbols) and $SrFe_{7n/8}M_{n/8}O_{2.875}$ (green symbols). The different oxygen sites are indicated by asterisks (O1), triangles (O2) and circles (O3). For the early transition metals, the O p-band center values have a strong dependence on the location of the oxygen vacancy, so that the combined effect of dimensionality and composition spans an energy interval of 0.3 eV. The influence of composition/structure diminishes when moving to the right of the 3d series.

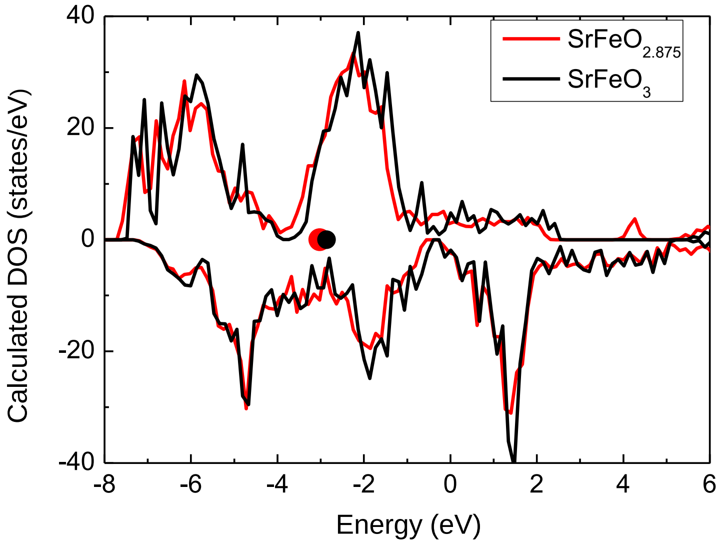

Figure S5. Calculated density of states for $SrFeO_3$ (black) and $SrFeO_{2.875}$ (red). The Fermi level is set as the zero of energy. Up spin (or majority) and down spin (or minority) contributions are shown. DOS values are given per calculated cell. The black and red circles denote the O p-band center of $SrFeO_3$ and $SrFeO_{2.875}$, respectively.

Table S2. O p-band centers and predicted surface exchange coefficient according to the linear relationship found by Morgan *el at*. [1] Log k* = 3.3458*(Op-band center) +1.7543. Note that this linear relationship was extracted using O p-band centers evaluated in the $E_{max}\rightarrow\infty$ limit.

Compound	O p-band center (eV) at $E_{max}\rightarrow\infty$	Surface exchange coefficient k* (cm/s)	O p-band center (eV) at E_{max} = 4 eV	Surface exchange coefficient k* (cm/s)
$SrFeO_3$	-1.54	4.00 10^{-4}	-2.86	1.53 10^{-8}
$SrFeO_{2.875}$	-2.02	9.90 10^{-6}	-3.09	2.60 10^{-9}
$SrFe_{7/8}Ti_{1/8}O_3$	-1.93	1.98 10^{-5}	-2.83	1.93 10^{-8}
$SrFe_{7/8}Ti_{1/8}O_{2.875}$	-1.91	2.31 10^{-5}	-2.95	7.66 10^{-9}
$SrFeNiO_{2.875}$	-1.84	3.96 10^{-5}	-2.89	1.22 10^{-8}
Sr_2FeO_4	-1.99	1.25 10^{-5}	-2.95	7.66 10^{-9}
$Sr_3Fe_2O_7$	-1.78	6.29 10^{-5}	-2.83	1.93 10^{-8}

[1] R. Jacobs, T. Mayeshiba, J. Booske, and D. Morgan, *Adv Energy Mater* **2018**, *8* (11), 1702708.
